# Case report: Klinefelter syndrome may protect against the development of spinal and bulbar muscular atrophy

**DOI:** 10.3389/fneur.2024.1340694

**Published:** 2024-02-09

**Authors:** Haruna Akanuma, Suguru Kadowaki, Kazuaki Kanai

**Affiliations:** ^1^Department of Neurology, Fukushima Medical University School of Medicine, Fukushima, Japan; ^2^Department of Neurology, Ohta General Hospital Foundation, Ohta Nishinouchi Hospital, Kōriyama, Japan

**Keywords:** testosterone, spinal and bulbar muscular atrophy, Klinefelter syndrome, androgen replacement therapy, leuprorelin

## Abstract

Spinal and bulbar muscular atrophy (SBMA) is an X-linked recessive motor neuron disease caused by the expansion of cytosine-adenine-guanine (CAG) repeats in the androgen receptor (AR) gene. It is thought that the nuclear translocation of abnormal AR proteins following binding to testosterone triggers the onset of the disease. We report the case of a patient who had SBMA coincident with Klinefelter syndrome. He developed SBMA symptoms rapidly after receiving androgen replacement therapy for Klinefelter syndrome. No cases of coincident SBMA and Klinefelter syndrome have been reported, and if confirmed by further patients in future, that androgen hormones are strongly associated with the development and progression of SBMA in fact in humans.

## Introduction

Spinal and bulbar muscular atrophy (SBMA) is an X-linked recessive motor neuron disease caused by the expansion of CAG repeats in the androgen receptor (AR) gene (1). It is thought that the nuclear translocation of abnormal AR proteins following binding to testosterone triggers the onset of the disease (2). Suppression of testosterone by luteinizing hormone-releasing hormone (LHRH) analogs can disrupt the pathomechanisms of the disease (3), and it has been used clinically as a treatment for SBMA (4). Klinefelter syndrome is caused by a supernumerary X chromosome in an XY male as a result of the meiotic nondisjunction of the X chromosome (5). Males with Klinefelter syndrome often have decreased serum testosterone concentrations and elevated luteinizing hormone and follicle-stimulating hormone levels. Testosterone supplementation is used to treat some symptoms of Klinefelter syndrome that result from abnormal hormone levels (6). Here, we present a patient who had Klinefelter syndrome who developed SBMA symptoms rapidly after receiving androgen replacement therapy.

## Case presentation

**Figure 1:**
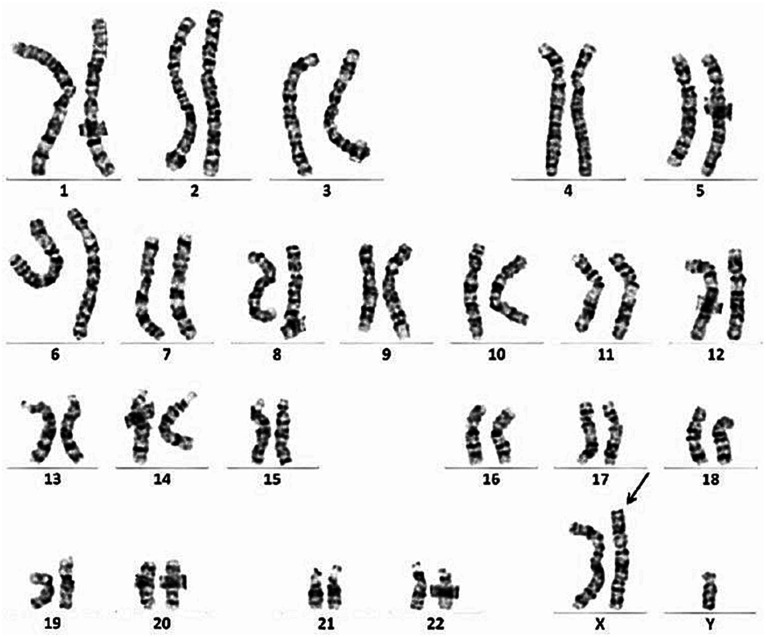
Chromosome analysis reveals an abnormal karyotype 47, XXY.

The patient was a 49-year-old man. At the age of 38, he developed muscle spasms and muscle twitching in the extremities and abdomen. He visited a neurology clinic at the age of 40, at which time stiff-person syndrome was initially suspected. Although a thorough examination was performed, the cause was undetermined. At that visit, his serum creatine kinase (CK) levels were within normal limits (221 IU/L, reference value: 62–287 IU/L). At the age of 46, he complained of infertility and was examined by a urologist. He was found to have severe atrophy of the testes as well as azoospermia. Chromosome analysis revealed an abnormal karyotype 47, XXY and he was diagnosed with Klinefelter syndrome (Figure 1). His serum total testosterone level at this time was 295.3 ng/mL (reference value: 142.4–923.1 ng/mL), which was at the lower limit of normal. However, his luteinizing hormone level was 21.89 mIU/mL (reference value: 0.1–8.7 mIU/mL) and follicle-stimulating hormone level was 23.95 mIU/mL (reference value: <0.3–13.8 mIU/mL), which were both above the upper limit of normal. Thus, androgen replacement therapy was initiated to treat Klinefelter syndrome. At the age of 48, he developed postural instability and tremors in the upper limbs. He visited the neurology clinic again, at which time Parkinson’s disease was suspected and a thorough examination was performed; however, there were no notable abnormalities other than an elevated serum CK level of 487 U/L. Shortly thereafter, his knees began to buckle more frequently, and he could no longer climb stairs without a handrail. The frequency of muscle spasms also increased and occurred daily. He visited our department for a detailed examination at age 48.

**Figure 2:**
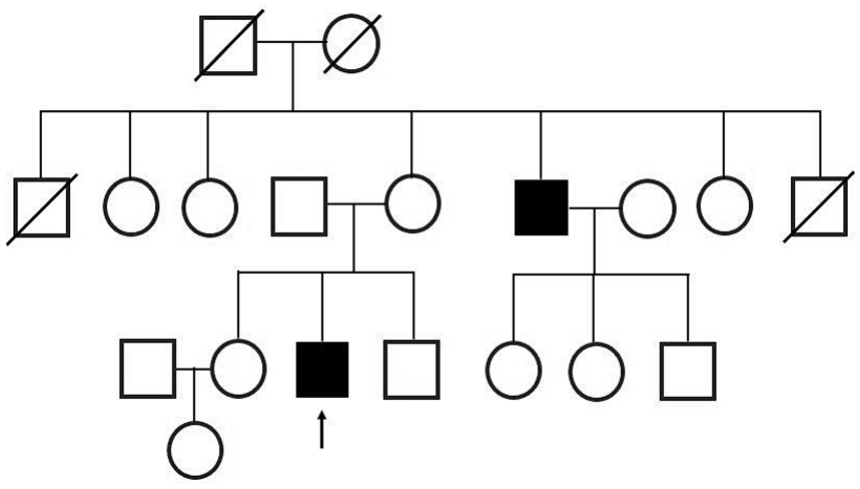
Family tree. The patient’s maternal uncle was diagnosed with SBMA.

At the time of his visit to our department, physical and neurological examinations indicated gynecomastia, tongue atrophy, postural tremors in both upper extremities, proximal muscle weakness, and loss of tendon reflexes in the extremities. He complained of subjective muscle twitching, but no fasciculation was visible. During this initial visit, we noticed that he had a family history of SBMA: his maternal uncle was diagnosed with SBMA at age 47 and was treated with LHRH analogs (Figure 2).

**Figure 3:**
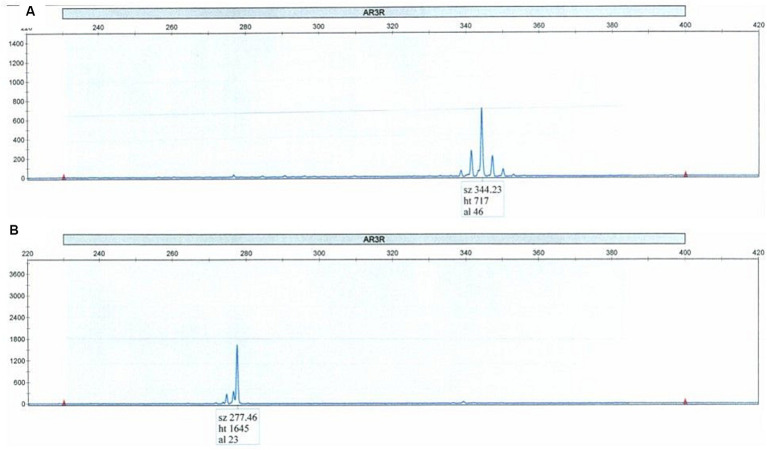
Polymerase chain reaction test using capillary electrophoresis **(A)** shows the expansion of 46 CAG repeats. Only one peak was detected, indicating that both alleles of the AR gene had the same expanded CAG repeats. Normal control is shown in the lower panel **(B)**.

Blood tests revealed an elevated serum CK level of 610 IU/L (reference value: 59–248 IU/L), and total testosterone level of 6.45 ng/mL (reference value: 1.71–8.71 ng/mL). Nerve conduction studies showed a decreased amplitude of sensory nerve action potentials and needle electromyography showed a markedly increased amplitude of motor unit potentials. Electrocardiography showed Brugada-type ST-segment elevation. Genetic testing revealed expanded CAG repeats (*n* = 46) in the AR gene, and he was diagnosed as a coincidental case of SBMA and Klinefelter syndrome. Only one peak was detected in the polymerase chain reaction test using capillary electrophoresis, indicating that both alleles of the AR gene had the same expanded CAG repeats (Figure 3).

**Figure 4:**
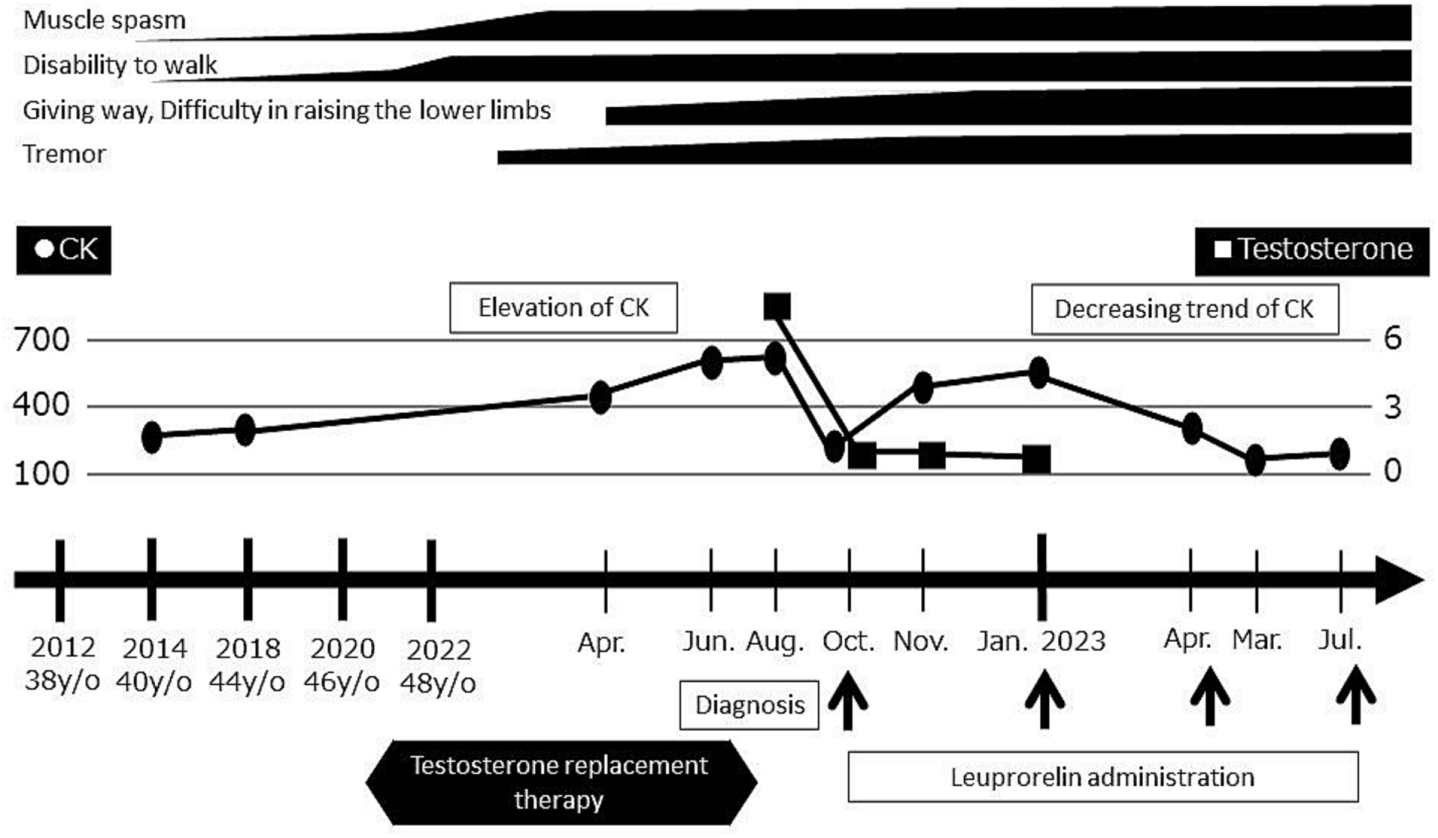
Timeline of evaluations and diagnosis. Serum CK levels and motor symptoms worsened after the initiation of androgen replacement therapy. Upon the discontinuation of androgen replacement therapy and initiation of leuprorelin, the serum CK levels decreased and symptoms became stable.

Shortly thereafter, androgen replacement therapy was discontinued and the LHRH agonist leuprorelin was started for the treatment of SBMA. Since that time, his symptoms have remained stable, his CK levels have trended downward, and his testosterone is now at a castrate level (Figure 4).

## Discussion

SBMA (1) is thought to be caused by a gain-of-function mutation in the AR gene, which results in mutant AR proteins binding to testosterone and translocating from the cytoplasm to the nucleus, where these mutant proteins accumulate and induce cytotoxicity in motor neurons and skeletal muscle (2, 7). It is thought that the binding of testosterone to the AR protein is necessary for the onset of SBMA (2). Thus, women with an AR gene with expanded CAG repeats do not develop the disease and are asymptomatic because their testosterone levels are much lower than those of men. It was reported that even women who are homozygous for an AR gene with expanded CAG repeats have only minor symptoms and do not develop SBMA (8). The administration of testosterone in a transgenic mouse model was shown to trigger the development of SBMA (2). To the best of our knowledge, no direct evidence of this mechanism of action has been shown in male patients with SBMA or carrier women with an AR gene with expanded CAG repeats.

As far as we are aware, no cases of coincident SBMA and Klinefelter syndrome have been reported (9). We hypothesize that the androgen replacement therapy for Klinefelter syndrome may have triggered the development of SBMA. The patient had relatively low serum androgenic hormone levels before starting androgen replacement treatment, probably as a result of Klinefelter syndrome. In addition, tremor is the first and most frequent symptom in male patients with SBMA, but less frequent in female patients (10). In the current patient, only mild muscle spasms and muscle twitching were observed before the initiation of androgen replacement therapy, which is similar to the symptoms observed in female carriers (8, 11). Furthermore, although it was reported that a decrease in serum creatinine levels precedes the onset of motor symptoms by approximately 15 years in male patients with SBMA, no decrease in serum creatinine levels was observed in this patient. After administrating testosterone, this patient developed tremors and weakness, which resembled the results shown in a transgenic mouse model (2). This suggests that testosterone induces the development of subclinical SBMA in patients with Klinefelter syndrome.

In this case, the protective effect of Klinefelter syndrome against the development of SBMA may be explained by low testosterone levels suppressing the translocation of AR to the nucleus, rather than owing to the low testosterone levels *per se*. This is because a previous case report showed that a transgender patient with SBMA who had been using spironolactone for a long time to achieve gender reassignment developed SBMA despite having low levels of serum testosterone (12). In that report, it was discussed that the action of spironolactone as a partial agonist or selective AR modulator led to the development of SBMA. The results of these two case reports are consistent with those of a study that showed the efficacy of LHRH analogs and the ineffectiveness of androgen antagonists in an SBMA mouse model (13). This is interesting because it demonstrates that the pathology of the human disease and that of the mouse model are consistent.

The karyotype of this patient was 47, XXY and the AR gene was located on the X chromosome indicating this patient had two alleles of the AR gene with abnormally expanded CAG repeats; thus, the expression level of the abnormal AR protein may also be high. Therefore, the initiation of testosterone replacement therapy in this patient may have caused an earlier and more severe onset of symptoms by promoting a higher degree of nuclear translocation of the abnormal AR protein bound to testosterone. Although it was reported that excess X chromosome inactivation occurs in the majority of cells in Klinefelter syndrome (11, 12), 15%–30% of the genes on the inactivated X chromosome escape inactivation (13). At this time, the extent to which the AR gene is expressed or inactivated in patients with Klinefelter syndrome is unknown. Further cell biological studies will be necessary to examine this problem.

To the best of our knowledge, this is the first report of a patient who had coincident SBMA and Klinefelter syndrome and provides the first direct evidence that testosterone triggers SBMA in patients. This case provides new and interesting insights into the role of testosterone in the pathomechanism of SBMA development.

## Data availability statement

The raw data supporting the conclusions of this article will be made available by the authors, without undue reservation.

## Ethics statement

Written informed consent was obtained from the individual for the publication of any potentially identifiable images or data included in this article.

## Author contributions

HA: Writing – original draft. SK: Writing – review & editing. KK: Writing – review & editing.
